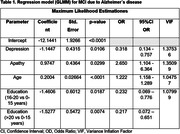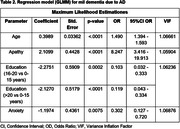# A predictive regression model for mild cognitive impairment and Alzheimer´s disease using real‐world electronic health records

**DOI:** 10.1002/alz.086757

**Published:** 2025-01-09

**Authors:** Raquel Yubero, Rocio García Cobos, Elena Garcia Arcelay, Alicia Algaba, Pablo Rebollo, Jorge Mauriño, Rafael Arroyo

**Affiliations:** ^1^ Neurology Department, Hospital Quirónsalud Madrid, Madrid Spain; ^2^ Neurology Department, Hospital Quirónsalud Madrid, Madrid, Madrid Spain; ^3^ Roche Farma S.A, Madrid, Madrid Spain; ^4^ IQVIA, Madrid, Spain, Madrid, Madrid Spain

## Abstract

**Background:**

Identifying individuals with early Alzheimer’s disease, i.e. mild cognitive impairment (MCI) and mild dementia due to Alzheimer’s disease (AD) can facilitate the rapid initiation of pharmacological and non‐pharmacological therapies with the aim of achieving better clinical outcomes. However, an early and accurate diagnosis is still limited in clinical practice.

This study aimed to identify factors associated to MCI and AD using a real‐world dataset from a memory clinic in Spain.

**Methods:**

This non‐interventional, case‐control study used the Quirónsalud Madrid Database, which includes information from people who were seen for cognitive problems from 2007 to 2022.

The dataset incorporated variables such as the Global Deterioration Scale (GDS), Neuropsychiatric Inventory Questionnaire (NPI), demographic characteristics such as age, educational level, profession, vascular risk factors (hypertension, diabetes mellitus, smoking), family history of dementia, alcohol consumption, and neurological diagnosis based on the International Classification of Diseases.

Two regression models were built as Generalized Linear Mixed Models (GLMM): 1) 644 patients with MCI (GDS = 3) vs. 623 controls (GDS≤2); 2) 966 patients with AD (GDS≥3 and AD diagnosis) vs. 623 controls.

**Results:**

According to GLMM, variables associated to MCI were age [OR = 1.222], apathy [OR = 2.650], depression [OR = 0.318] and education [OR = 0.232 (16‐20 years) and 0.217 (>20 years) vs. <15 years] (table 1) with an AUC of the ROC curve of 0.657, sensitivity of 0.82 and specificity of 0.45. In the GLMM adjusted for AD, variables included were age [OR = 1.490], family history [OR = 4.147 (first degree vs none)], apathy [OR = 8.247], anxiety [0.302], and education [OR = 0.103 (16‐20 years) and 0.119 (>20 years) vs. <15 years] (table 2) with an AUC of the ROC curve of 0.852, sensitivity of 0.84 and specificity of 0.73. No association with vascular risk factors was found.

**Conclusions:**

Age, education, and apathy were consistently associated with both MCI and AD. The AD constructed model demonstrated good classification performance, providing insights for diagnosis. Further studies are needed to develop a simple tool for MCI and AD detection.